# Central IGF1 improves glucose tolerance and insulin sensitivity in mice

**DOI:** 10.1038/s41387-017-0002-0

**Published:** 2017-12-19

**Authors:** Hao Hong, Zhen-Zhong Cui, Lu Zhu, Shu-Ping Fu, Mario Rossi, Ying-Hong Cui, Bing-Mei Zhu

**Affiliations:** 1 Key Laboratory of Acupuncture and Medicine Research of Ministry of Education, Nanjing University of Chinese Medicine, Nanjing, Jiangsu 210023 P. R. China; 2 Regenerative Medicine Research Center, West China Hospital, Sichuan University, Keyuan Road 4, Gaopeng Street, Chengdu, Sichuan 610041 P. R. China; 3 Molecular Signaling Section, Laboratory of Bioorganic Chemistry, National Institute of Diabetes and Digestive and Kidney Diseases, Bethesda, MD 20892 USA

## Abstract

Insulin-like growth factor 1 (IGF1) is a key factor for tissue growth and fuel metabolism. The potential function of central IGF1 remains unclear. We previously observed that IGF1 expression is increased in the hypothalamus of obese mice lacking STAT5 in the central nervous system (CNS). In this study, we explored the potential metabolic function of central IGF1 by intracerebroventricular (ICV) injection of IGF1, over-expression of central IGF1 by administering an adeno-associated virus (AAV), and ICV injection of an anti-IGF1 antibody. Mice that over-expressed central IGF1 displayed increased appetite, improved glucose tolerance and insulin sensitivity, decreased *Pomc* levels in the hypothalamus, and increased UCP1 expression in brown fat tissue. This is the first study demonstrating that central IGF1 regulates several important metabolic functions.

## Introduction

Insulin-like growth factors (IGFs) regulate a very large number of important physiological processes. Insulin-like growth factor 1 IGF1 regulates seed somatomedin C, is adin C, is a factors (IGF*Igf*1 gene that has a similartors (IGFs) regulate a very lar^1^. IGF1 has multiple biological effects, including promoting cell growth and proliferation^2^ and regulating fuel metabolism peripherally^3^. IGF1, which acts through IGF1 receptors and/or hybrid insulin/IGF1 receptors, has significant amino acid sequence homology with insulin, and enhances insulin sensitivity in both animal models and human subjects^4^. IGF1 is primarily secreted by the liver and mediates the endocrine actions of growth hormone (GH). GH, the main regulator of circulating IGF1 level in mammals, has an intricate regulatory relationship with IGF1. Mice lacking IGF1 in the liver have shown to display enhanced insulin sensitivity and glucose homeostasis^5^. A recent study has found that IGF1 is produced by all cell types in the brain^6^. IGF1 plays an important role in childhood growth and continues to havee brainrowth hormone (GH). GH, the main reF1 production can be retarded by under-nutrition, growth hormone insensitivity, lack of growth hormone receptors, or failure of downstream signaling pathways including SHP2 and STAT5B (signal transducer and activator of transcription 5B)^7, 8^. Low serum IGF1 levels are associated with reduced insulin sensitivity, metabolic syndrome, glucose intolerance, and the development of type 2 diabetes^9^.

The potential role of central IGF1 in regulating whole body energy homeostasis has not been investigated. To address this issue, we sought to study the effects of central IGF1 in mice. Mice that lack *Stat*5a/b in the central nervous system (*Stat*5NKO mice) develop severe obesity, accompanied by hyperphagia, hyperleptinemia, impaired thermal response to the cold, and insulin resistance^10^. We recently completed RNA-seq studies showing increased expression of members of the *Igf* family, including *Igf*1*, Igf*2, *Igfals*, and *Igfbps*, in the hypothalamus of *Stat5*NKO mice. *Igf*1 expression was very low in wild-type mice but elevated 8-fold in the *Stat5*NKO mice (Figure S1A). To determine the potential function of central IGF1 in energy metabolism, we subjected wild-type mice to ICV injections of IGF1 and an anti-IGF1 antibody. We found that IGF1 increased food intake while the anti-IGF1 antibody decreased appetite. We also over-expressed IGF1 long-term in the brain of wild-type mice by administering ICV injections of a recombinant adeno-associated virus 2 (AAV2). The phenotypes that we observed with the virus-treated mice were similar to those observed after acute ICV IGF1 injection.

## Materials and methods

### Generation of the IGF1 over-expression construct

The AAV2 construct encoding *Igf*1 (AAV-*Igf*1*;* Figure S2A) was generated by using a genomic 7.4 kb fragment containing all *Igf*1 exon sequences of the murine *Igf*1 gene. The flag–tag followed the IGF1-promoter fragment after the restriction enzyme was cut. AAV packaging was performed by the University of Pennsylvania Vector Core (Lot: V4738MI-S).

### Animals and metabolic measurements

Male C57BL/6J mice (8–12 weeks old) were handled and housed in the Experimental Animal Center, located in building 14C at the National Institutes of Health, Bethesda, Maryland. This study was approved by the Institutional Animal Care and Use Committee of National Institute of Diabetes, Digestive and Kidney Diseases (NIDDK, K005-LBC-15), and all procedures were conducted in accordance with the guidelines of the National Institute of Health Animal Care and Use Committee. Mice were randomly (using a computerized, random number generator through the block-randomization method of Statistics Analysis System version 9.2.) divided into six groups: saline and IGF1 group, saline and anti-IGF1 group, AAV-empty and AAV-*Igf*1 injection group. Food intake (averaged per day), and body weight were measured daily after injection in each group. A total number of animals should be able to show a desired effect as few or many as possible (five animals in each group is required at least^11^). As our reaserch was a pilot study, so according to the reduced requirement (the number of animals used in experiment) from U.S. law and animal experimentation: 10 mice were included in each group for our experiment to avoid unnecessary suffer. In vivo metabolic tests were performed using standard procedures. To measure glucose tolerance (IGTT), mice were fasted overnight for 12 h, and blood was collected from the tail vein immediately before and 15, 30, 60, 90, and 120 min after i.p. injection of glucose (2g/kg) to determine blood glucose concentrations. To obtain a measure of peripheral insulin sensitivity (ITT), mice were fasted for 4 h, and blood glucose levels were measured before and at the indicated time points after i.p. injection of human insulin (0.75 U/kg). Blood glucose levels were determined using an automated blood glucose reader (Glucometer Elite Sensor; Bayer). Plasma insulin concentrations were determined by using an ELISA kit (Crystal Chem Inc. Cat.No.:#90080).

### AAV-*Igf*1, IGF1 & anti-IGF1 injection

Pre-experiments were carried out for each injection before the formal study. Only those mice that recovered from the surgery could be allocated to experimental groups, others were euthanized according to the guidelines of the National Institute of Health Animal Care and Use Committee.

Mice were injected with 2 ul (1 ug/ml) of IGF1 (Peprotech, Catalog #: 100-11) or an anti-IGF1 antibody (Abcam, ab9572), or with 10^11^ GC/mouse of the AAV-*Igf*1 virus into the arcuate nucleus of the hypothalamus (ARC). Following injections, mice were individually housed, and body weight and food intake were determined as described above.

Mice were anesthetized with ketamine and xylazine (100–200 mg/kg and 5–10 mg/kg, respectively, given i.p.) and placed on a heating pad during anesthesia, surgery, and recovery. The mice were placed in a small-animal stereotaxic instrument (head held with ear bars and incisor holder). The surface of the skull were exposed with sterile surgical instruments then opened with a small burr drill over the appropriate stereotaxic coordinates. The dura was opened with a fine needle. A 30 gauge stainless steel needle attached to a 5-ul Hamilton syringe was lowered into the burr hole and 500 to 2 ul of the preparation was administered over 3–5 min per site (ARC). The animal was returned to its cage and monitored carefully twice daily for the first 48 h. Post-operative analgesia consisted of ketoprofen (5 mg/kg i.m. or s.c.) given every 24 h. A plastic cap was placed at the top of the cannula and removed for each injection after a 1-week recovery.

### Brain immunofluorescence staining and liver histology

Twelve days after injection, the mice brains were collected and stored in 4% PFA overnight. Brains were sectioned at 40 um in thickness with Leica VT1000S. Sections were collected for the entire hypothalamus. Three sets of brain sections were stored at 4 °C in paraformaldehyde/phosphate-buffered saline (PBS). Flag antibodies: flag (F1804 sigma, 1:300, Secondary antibodies: Life Technology, A11017 Goat anti mouse 488; NeuN (MAB377, 1:1000); Secondary antibodies: Life technology, A11020, Goat anti Mouse 594; GFAP(C9205, 1:1000), Fluor conjugated 594 antibody, IGF1(abcam ab9572, 1:1000), secondary antibody Life technology, A11008, (1:1000)), Goat anti rabbit 488, and abcam Ab150064 (1:1000), donkey anti rabbit 594. Data analysis: fluorescent images were taken with NIDDK ALMIAC Keyence Digital Microscope and captured under exactly the same condition for both AAV-GFP (empty) control virus-injected and AAV-*Igf*1 virus-injected mice. Fiji free software (formerly image J, NIH) and Fiji program were used to run “MeanSigPerPixel_ROI-bg.ijm” Macro (For details, see word format text in 4th supplementary Material). Images were processed as original TIFF images after background deduction. Average total pixels were obtained for AAV-GFP (empty) control images and AAV-*Igf*1 images.

Mouse liver was harvested and fixed in 4% paraformaldehyde/phosphate-buffered saline for overnight and embedded in paraffin. For each liver, 5 um-thick sections were mounted on slides and stained with hematoxylin and eosin (HE) staining. Image acquisitions were performed using a BZ-II Viewer.

### RNA and protein analyses

Total RNA was isolated using Trizol reagent (Cat. No.: 15596018) and QIAGEN RNase kit (Cat .No.: 74104). RNA concentrations were determined by a Qubit bio-analyzer (QUBIT2.0, Invitrogen, Q32866) prior to real-time PCR for gene expression. Western blotting was carried out with lysates prepared from isolated mouse liver or BAT by using standard techniques. Protein bands were quantitated using Image J software. The primers and antibodies used are listed below (See supplemental Tables 1 & 2).

### Measurement of hepatic glycogen and triglyceride levels

Liver samples were homogenized in PBS, and glycogen and triglyceride contents were detected by using commercially available kits (Sigma; Cat. No.: MAK016 and Cat. No.: TR0100, respectively), strictly following the manufacturer’s instructions.

### Statistical analysis

Statistical analyses were performed using SPSS 18.0. Multiple group comparisons were made with ANOVA, followed by the Tukey HSD test for multiple comparisons. Comparisons between two groups were performed using unpaired 2-tailed Student’s t test, and rank sum test was used for more than two time points. Data are shown as means ± SD, *P* < 0.05 was considered statistically significant.

## Results

### Hypothalamic IGF1 expression is increased in obese mice

We first examined IGF1 protein expression in the hypothalamus of the *Stat*5NKO mice. We found a higher IGF1 expression in *Stat*5NKO mice compared to their *Stat*5fl/fl lean control littermates (Figure S1B). To test the hypothesis that IGF1 expression is increased in other obese mouse-models, we also studied mice that had been maintained on a high-fat diet. In this model of diet-induced obesity (DIO), hypothalamic IGF1 expression was also significantly increased, compared to mice fed a standard chow diet (CD) (Figure S1C).

### Over-expression efficiency of *Igf*1 in the arcuate nucleus of hypothalamus

**Fig. 1:**
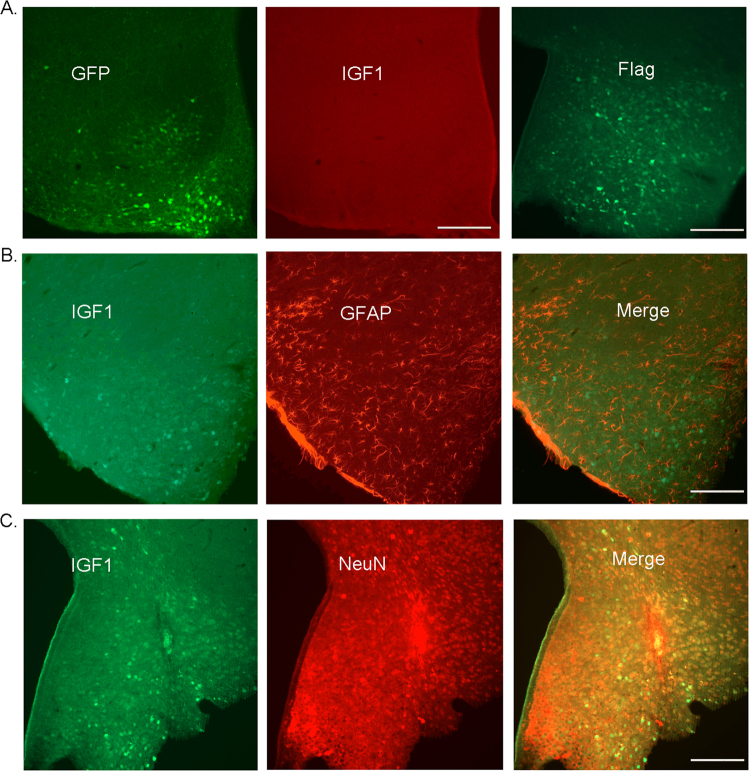
Immunostaining to detect AAV-GFP(empty) and AAV-IGF1 expression **a** Representative immunofluorescence staining of GFP fluorescence and IGF1 to show viral expression in the hypothalamus of AAV-GFP (empty) injected mice. **b** Representative immunofluorescence staining to detect IGF1 expression and GFAP (Glial marker) expression in the hypothalamus of AAV-*Igf*1 injected mice. **c** Representative immunofluorescence staining to determine IGF1 and NeuN (neuron marker) expression in the hypothalamus of AAV-*Igf*1 injected mice. Scale bar indicates 100 um, *n* = 10

**Fig. 2:**
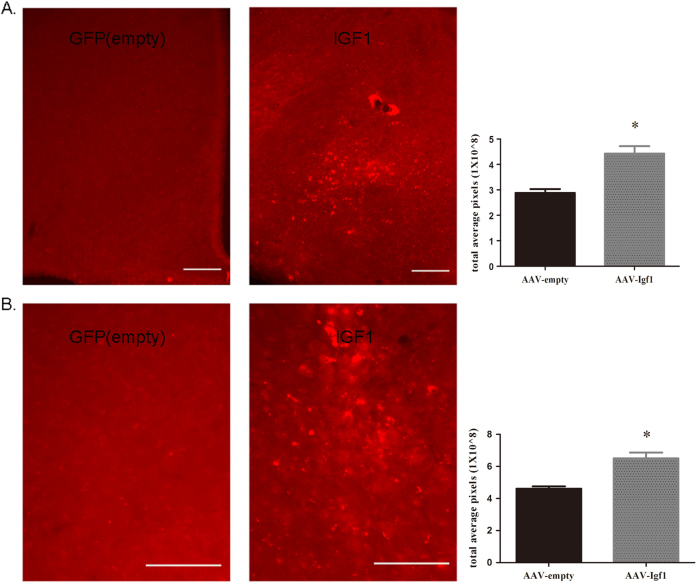
Compare IGF1 expression in the hypothalamus of AAV-GFP and AAV-*Igf*1 virus injection through immunostaining **a** IGF1 antibody staining and total-pixel calculation in both AAV-GFP- and AAV-IGF1-injected mouse brain sections (*n* = 10, scale bar = 100 um). **b** IGF1 antibody staining and total-pixel calculation in the hypothalamus of AAV-GFP- or AAV-*Igf*1-injected mice (*n* = 10, scale bar = 100 um). Data are shown as means ± SD, *P < 0.01 vs. AAV-GFP group

After injecting recombinant AAV virus into the arcuate nucleus (ARC, 10^11^ genome copies/mouse) of wild-type mice, IGF1 over-expression in the ARC was confirmed via immunofluorescence (Fig. 1a-c). Both A and B are stained sections of the arcuate nucleus to show virus expression through GFP fluorescence and IGF1 antibody staining in AAV-GFP (empty) injected mice (Fig. 1a). We then applied flag tag antibody-staining to show AAV-*Igf*1 virus-expression after AAV-*Igf*1 injection into the mouse brain. Both IGF1 and GFAP (Glial marker) expressions were detected via immunostaining in AAV-*Igf*1-injected mice. The stained astrocytes showed only GFAP expression, and no IGF1 (Fig. 1b). Furthermore, both IGF1 and NeuN (neuron marker) expression in AAV-*Igf*1-injected mice were observed via immunostaining (Fig. 1c). These results showed that most IGF1-positive cells were NeuN-positive as well, indicating the expression of injected AAV-*Igf*1 in neurons. We also examined for IGF1 over-expression efficiency after AAV-GFP and AAV-*Igf*1 virus injection in the hypothalamus of the mice using immunostaining and western blot. Our experiment showed that IGF1 expression increased significantly in the hypothalamus of AAV-*Igf*1 mice than that in the AAV-GFP-injected mice. Total pixel-calculation that followed also verified that IGF1-expression level was higher in the AAV-*Igf*1group than in the AAV-GFP group (Figs. 2a, b, Figure S2B). These results indicated that AAV-*Igf*1 injected mice had increased IGF1 expression in the brain, indicating a successful IGF1 over-expression model.

### Central IGF1 increases food intake

**Fig. 3:**
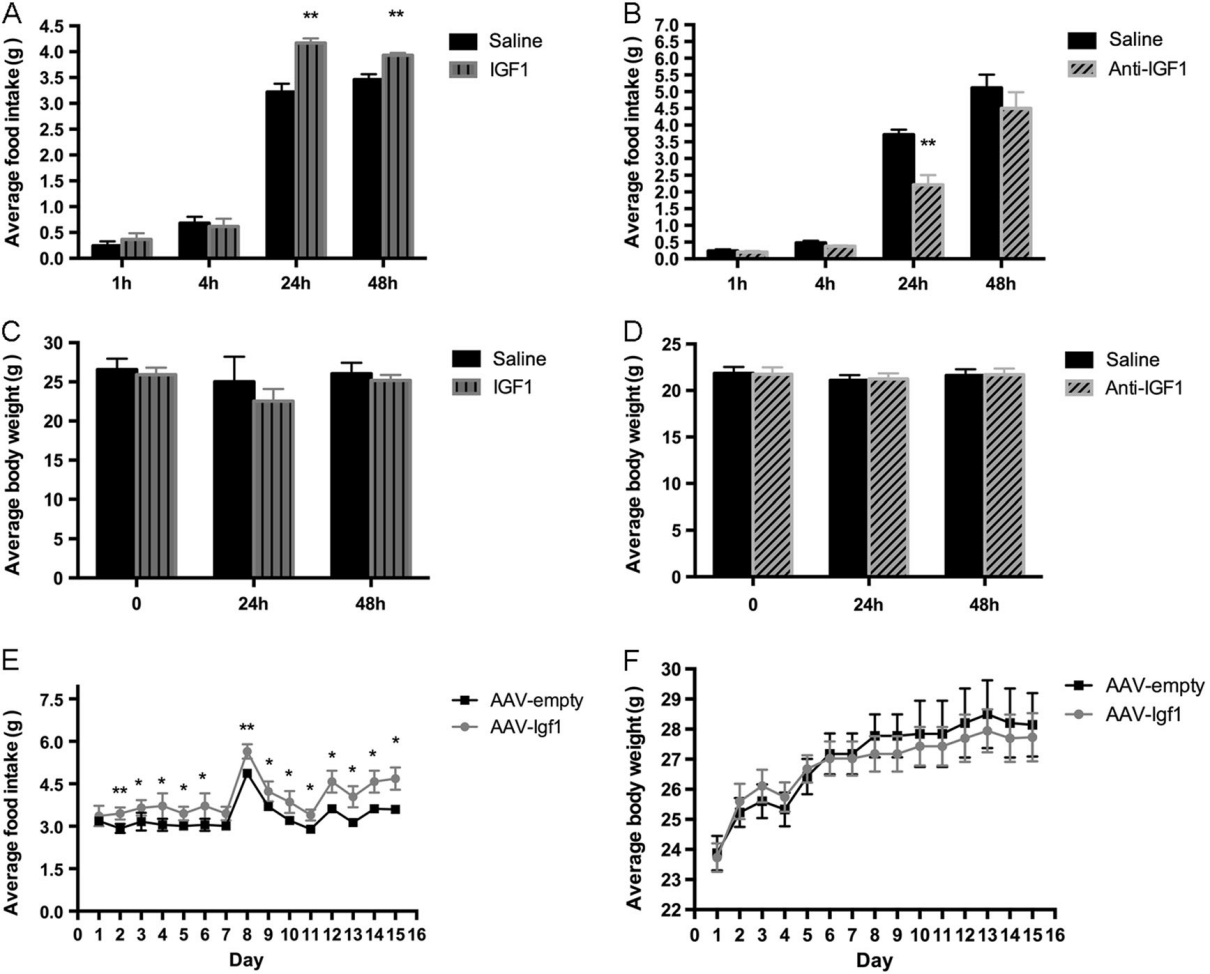
IGF1 promotes food intake in wild-type mice IGF1 or an anti-IGF1 antibody were injected ICV (2 ul, 1 ug/ml). AAV-*Igf*1 was injected into the ARC (10^11^ GC/mouse) of wild-type mice. **a** & **b** Food consumption 48 h after IGF1 and anti-IGF1 injection, *n* = 10 per group. **c** & **d** Body weight 48 h after IGF1 and anti-IGF1 injection, *n* = 10 per group. (**e** & **f**) Food consumption and body weight 2 weeks after virus injection, *n* = 12 per group. Data are expressed as means ± SD. *P < 0.05, **P < 0.01 vs. saline or AAV-GFP group

To explore the potential function of central IGF1, we injected IGF1 or an anti-IGF1 antibody into the lateral cerebral ventricle (ICV) of wild-type mice. The IGF1-injected mice displayed a significant increase in food intake (Fig. 3a), where as the mice injected with the anti-IGF1 antibody showed reduced food intake (Fig. 3b). Neither treatment affected body weight during the 48-hour observation period (Figs. 3c, d). To study the effects of long-term IGF1 over-expression in the brain, we injected the wild-type mice with a recombinant AAV coding for IGF1. Specifically, the virus was injected into the ARC, which plays a key role in the central regulation of food intake and energy homeostasis. We found that the virus-treated mice displayed enhanced appetite but unchanged body weight (Figs. 3e, f), mimicking the effects of acute central IGF1 administration.

### Central IGF1 improves glucose homeostasis and insulin sensitivity

**Fig. 4:**
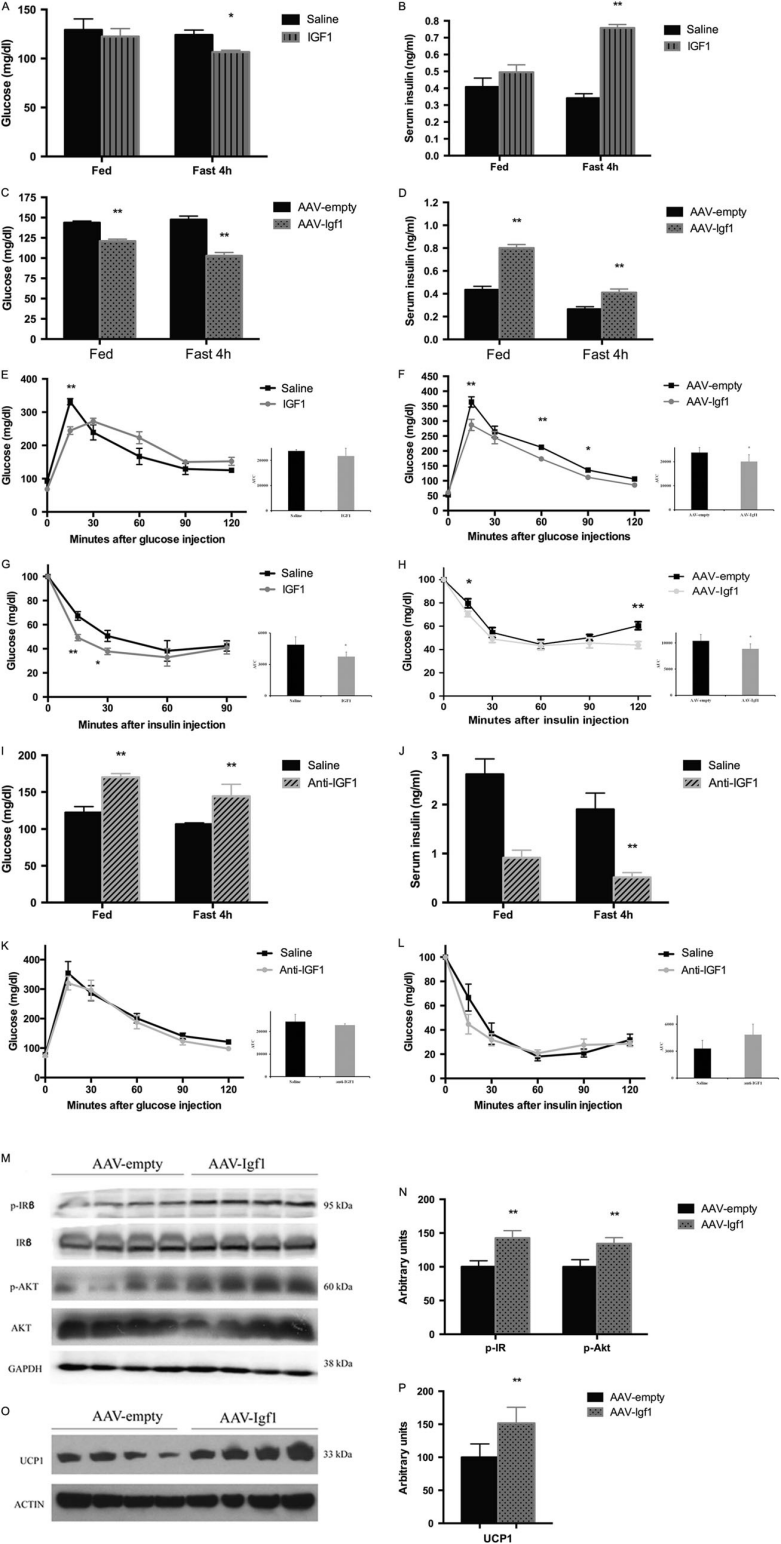
Effect of central IGF1 on various metabolic parameters in wild-type mice . Glucose and insulin responses were measured after ICV injection of IGF1 or anti-IGF1, or ARC injection of AAV-*Igf*1 injection. **a** & **b** Free-fed and fasting blood glucose and serum insulin levels after IGF1 injection. (**c** & **d**) Free-fed and fasting glucose and serum insulin levels after AAV-*Igf*1 virus injection. **e** &** f** Glucose tolerance tests (GTT) after IGF1 or AAV-*Igf*1 injection. Glucose (2 g/kg) was administered i.p after a 14 h fast (mouse age: 13 weeks). **g** & **h** Insulin tolerance tests (ITT) after IGF1 or AAV-*Igf*1 injection. Insulin (0.75 U/kg) was administered i.p. after a 4 h fast (mouse age: 12 weeks). **i** & **j** Free-fed and fasting (4 hr fast) blood glucose and serum insulin level after anti-IGF1 injection. **k** & **l** GTT and ITT after anti-IGF1 injection. (m) Immunoblots of insulin receptor β subunit and Akt in liver after AAV-*Igf*1 virus injection. **n** Ratio of p-Akt, p-IR β in liver of AAV-*Igf*1 mice relative to AAV-GFP mice (quantitative analysis was normalized by total IR or total Akt). **o** Immunoblots of UCP1 expression in brown adipose tissue (BAT) after AAV-*Igf*1 virus injection. **p** Ratio of UCP1 expression in BAT of AAV-*Igf*1 mice relative to AAV-GFP mice. Data are repeated twice and expressed as means ± SD. *P < 0.05, **P < 0.01 vs. saline or AAV-GFP group, *n* = 10

We then measured serum insulin and blood glucose levels in mice fasted for 4 or 16 (o/n) hours. We found that acute central IGF1 treatment (Figs. 4a, b) or prolonged IGF1 over-expression in the ARC (Figs. 4c, d) significantly increased serum insulin and reduced blood glucose levels. Glucose and insulin tolerance tests showed that both treatments led to improved glucose tolerance (Figs. 4e, f) and enhanced insulin sensitivity (Figs. 4g, h). In contrast, ICV injection of the anti-IGF1 antibody caused decreased serum insulin and elevated blood glucose levels (Figs. 4i, j). Somewhat surprisingly, these mice displayed normal glucose tolerance and insulin sensitivity (Figs. 4k, l).

To explore the mechanism by which central IGF1 improved glucose homeostasis and insulin sensitivity, we examined the levels of Akt and the insulin receptor beta subunit (ISR-beta) in the liver, which are predicted to play a key role in IGF1 signaling^12–14^. Akt activation is associated with its auto-phosphorylation at Thr-473^15^. Western blotting studies demonstrated that the abundance of total ISR-beta and Akt did not differ between control and AAV-*Igf*1 mice, whereas the expressions of the phosphorylated (active) forms of Akt and ISR-beta were significantly increased in the AAV-*Igf*1 mice (Figs. 4m, n). These results provide further evidence that enhanced central IGF1 is critical for insulin responsiveness through activation of Akt and ISR-beta.

The presence of UCP1 in brown adipose tissue (BAT) directs its oxidative metabolism almost entirely to thermogenesis^16, 17^. UCP1/BAT activity is a well-known regulator of energy homeostasis. Western blotting studies showed that UCP1 expression in BAT from AAV-*Igf*1 mice was significantly increased, compared to control mice (mice injected with empty AAV) (Fig. 4o, p). Strong neuroanatomical and functional evidence has shown that WAT and BAT are innervated and regulated by the sympathetic nervous system (SNS) for thermoregulation and beige adipocyte formation^18^. Since UCP1 is the hallmark protein responsible for cold- and diet-induced thermogenesis in BAT^19^, its expression can also be regulated by SNS activity. These data suggest that central IGF1 action enhances central sympathetic outflow.

### Central IGF1 reduces liver weight and lowers triglyceride and glycogen levels

**Fig. 5:**
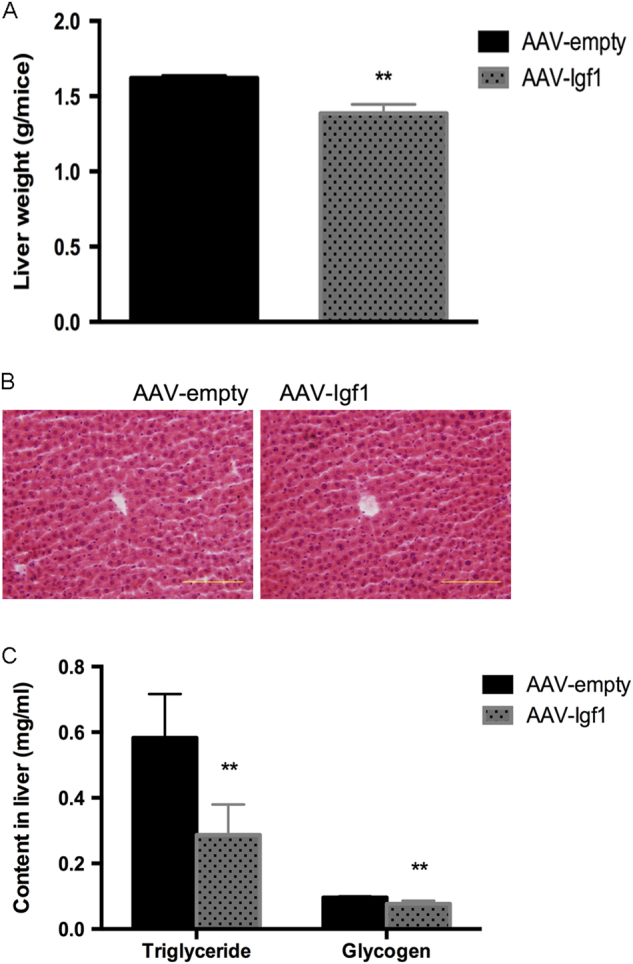
Central IGF1 reduces liver weight and lowers hepatic triglyceride and glycogen levels The AAV-*Igf*1 virus or AAV- GFP was injected into the ARC of wild-type mice. **a** Liver tissue was collected from 14-week-old male mice for weight measurements. **b** H&E staining of liver sections; scale bar, 100 um. **c** Liver triglyceride and glycogen content. Data are repeated twice and expressed as means ± SD. *P < 0.05, **P < 0.01 vs. AAV-GFP group, *n* = 10

We also examined the effects of long-term central IGF1 over-expression on liver weight and triglyceride and glycogen levels, and found that liver weight and hepatic triglyceride and glycogen levels were significantly reduced in AAV-*Igf*1 mice, compared to control mice (Figs. 5a, c). Liver morphology did not differ between AAV-*Igf*1 and control mice (Fig. 5b). These results suggest that central IGF1 signaling promotes glucose and lipid metabolism in the liver. This observation is consistent with the concept that central IGF1 enhances stimulates sympathetic outflow (see previous paragraph).

### Effects of IGF1 on gene expression of Pomc, Npy, Agrp in the hypothalamus

**Fig. 6:**
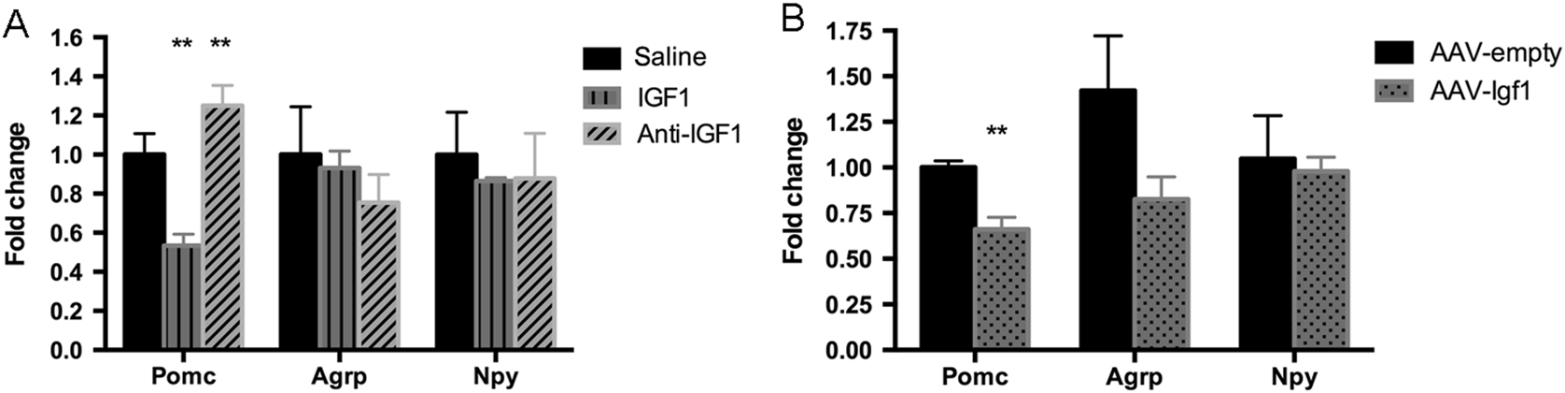
Expression of hypothalamic peptides regulating food intake after IGF1, anti-IGF1, or AAV-*Igf*1 injection . mRNA levels were determined via qRT-PCR. **a** *Pomc*, *Npy*, and *Agrp* expression after IGF1 or anti-IGF1 injection (ICV). **b** *Pomc*, *Npy*, and *Agrp* expression after AAV-*Igf*1 injection (into the ARC). Data are repeated twice and expressed as means ± SD. **P < 0.01 vs. saline or AAV- GFP group, *n* = 5

The ARC is a key hypothalamic nucleus in the regulation of appetite^20, 21^. Although the ARC contains other cell types, two main opposing types of output neurons have been defined: those expressing pro-opiomelanocortin (Pomc; Pomc neurons) and those containing Agouti-related protein (AgRP) and neuropeptide-Y (NPY) (AgRP neurons). The Pomc neurons release α-melanocyte stimulating factor (α-MSH), which collectively promotes activity and energy expenditure, and suppresses food intake. AgRP and NPY are neuropeptides that suppress energy use and promote food intake^22, 23^. To study whether central IGF1 modulates the expression of these appetite-regulating peptides, we used qRT-PCR to measure their expression levels after central administration of IGF1 or an anti-IGF1 antibody, or after long-term IGF1 expression in AAV-*Igf*1 mice. Acute IGF1 injection and long-term over-expression of IGF1 in the mouse brain did not affect *Agrp* and *Npy* expression, but significantly reduced Pomc expression (Figs. 6a, b). In contrast, central administration of the anti-IGF1 antibody increased Pomc expression, but did not change Agrp and Npy expression. It is therefore likely that the orexigenic effect observed after central IGF1 administration or over-expression is caused, at least partially, by a reduced Pomc expression.

## Discussion

The central nervous system plays an important role in the development of obesity^24, 25^. Loss of cytokine-STAT5 signaling in the CNS and pituitary gland alters energy balance and leads to obesity in mice^10^. We recently identified a large number of genes with altered expression levels in the hypothalamus of *Stat*5NKO mice (unpublished data). Surprisingly, in the hypothalamus of *Stat*5NKO mice, most members of the *Igf*1 family were found to be up-regulated. STAT5 has been shown to positively regulate IGF1 function in peripheral organs^26^, such as skeletal muscle and liver^27^. Some studies showed that modulating IGF1 has a dramatic effect on glucose metabolism in mice under both normal and high-fat diet feeding. For example, whole body over-expression of IGFBP1 effectively reduced the hypoglycemic effect of endogenous IGF1 and caused elevated plasma insulin levels, yet reduced insulin-stimulated glucose transport and glycogen synthesis in skeletal muscle, indicative of mild insulin resistance^28^. Pituitary-derived GH exerts its growth effects primarily by regulating the expression of IGF-I in both hepatic and non-hepatic tissues^29^. GH’s action is initiated upon binding to the cell-surface GH receptor (GHR), a homodimeric transmembrane protein. The GH-GHR interaction induces signal transduction through recruitment and activation of the cytosolic Janus kinase 2 (JAK2)^30^. The activated complex of signaling pathways includes four STAT pathways (STAT1, 3, 5a, and 5b), the MAPK (mitogen-activated protein kinase), and the PI3K (phosphoinositide-3 kinase) pathways. Activation of these pathways culminates in the regulation of multiple genes, including IGF1, IGFBP3, and IGFALS (acid labile subunit, ALS). One study has reported that brain-specific over-expression of IGFBP-6 under the control of glial fibrillary acidic protein promoter causes glucose intolerance, insulin resistance, and elevated weight gain both on a normal diet and on a high-fat diet^31^. Reduction of IGF1 signaling by inactivating the IGF1R gene in mice can also lead to impaired glucose homeostasis^32, 33^. However, these previous studies did not explore the potential role of IGF1 in the hypothalamus of the mouse brain. Given that *Stat*5 is a positive regulator of *Igf*1, *Stat*5 deficiency is expected to reduce *Igf*1 expression. However, we observed the opposite effect—an increase of *Igf*1 gene expression in the hypothalamus of *Stat5*NKO mice. As the central role of IGF1 remains controversial^34–36^, we performed the current study to explore the potential roles of central IGF1 in a more direct and systematic fashion.

In this study, we describe the metabolic phenotypes of IGF1- and IGF1 antibody-treated mice. Gain of function studies (ICV injection of IGF1 or AAV-mediated over-expression of IGF1) as well as loss of function experiments (ICV injection of an anti-IGF1 antibody) demonstrated that central IGF1 promoted food intake and insulin secretion, reduced blood glucose levels, and led to improved glucose tolerance (Fig. 4). Moreover, over-expression of IGF1 in the brain led to enhanced phosphorylation/activation of the insulin receptor (IRb subunit) and AKT, a key mediator of signaling pathways stimulated by insulin-like peptides^36^ (Fig. 4m). In addition, an increase in central IGF1 expression caused an increase of UCP1 expression in BAT (Fig. 4o), suggesting that central IGF1 can promote BAT activity and enhance energy expenditure. Since BAT activity is primarily under the control of the sympathetic nervous system, central IGF1 activity likely leads to an increase in central sympathetic outflow. Consistent with this notion, we also observed reduced hepatic triglyceride and glycogen levels (Fig. 5a-c). To the best of our knowledge, this is the first study that directly examined the role of central IGF1 in regulating lipid and glucose metabolism.

The regulation of energy homeostasis is closely controlled by the central nervous system and integrated signals from the periphery, includes insulin signaling^37^. Insulin acts on ARC neurons to inhibit the expression of the orexigenic neuropeptides NPY and AgRP and to enhance the expression of Pomc, an appetite-suppressing peptide, resulting in reduced food intake^38–41^. In contrast, we observed in the present study that over-expression of IGF1 in the ARC promoted food consumption, most likely due to an increase in Pomc expression. The receptors and neuronal pathways involved in this phenotype will be explored in the future studies. Meanwhile, the autonomic nervous system also plays a key role in the response to these signals, innervating peripheral metabolic tissues, including BAT, WAT, liver, and skeletal muscle^42^. Taking together, these new data shed light onto the role of central IGF1. In particular, the reported metabolic effects in BAT and in liver, caused by AAV-induced expression in brain of *Igf*1, are attributed mainly to a direct effect of IGF1 on neurons, and then mediated by the sympathetic nervous system. This is however highly speculative, and more experiments are required to justify this hypothesis.

Nevertheless, some limitations should be acknowledged in the interpretation of our results. One is that we only used single IGF1 and anti-IGF1 antibody doses. In addition, the detailed neuronal/cellular pathways through which central IGF1 exerts its metabolic actions remain to be explored. Also, the potential ICV IGF1 actions on other (non-hypothalamic) brain regions regulating food-intake need to be studied further. IGF2, which has similar homology with IGF1, is primarily secreted by the liver and in utero by the placenta. *Igf*2 has been reported to play an important role in the subventricular zone and in the subgranular zone of the hippocampus for adult-neurogenesis in mice^43^. Moreover, over-expression of *Igf*2 is associated with the intrauterine programming of adipose tissue and fetal overgrowth^44^. Current data about IGF2 and obesity is both limited and conflicting, so the potential role of central IGF2 in regulating energy and glucose homeostasis also need to be further elucidated. Nevertheless, our findings may guide future efforts to develop novel classes of drugs useful for the treatment of obesity and diabetes.

## Conclusion

In the present study, we systematically explored the role of central IGF1. We found that central IGF1 can promote feeding, improve insulin sensitivity and glucose tolerance, and stimulate energy expenditure via thermogenesis.

## Electronic supplementary material

Fig-suppl

Suppl-2
