# Correction to: Central IGF1 improves glucose tolerance and insulin sensitivity in mice

**DOI:** 10.1038/s41387-021-00153-4

**Published:** 2021-02-19

**Authors:** Hao Hong, Zhen-Zhong Cui, Lu Zhu, Shu-Ping Fu, Mario Rossi, Ying-Hong Cui, Bing-Mei Zhu

**Affiliations:** 1 Key Laboratory of Acupuncture and Medicine Research of Ministry of Education, Nanjing University of Chinese Medicine, Nanjing, Jiangsu 210023 China; 2 Molecular Signaling Section, Laboratory of Bioorganic Chemistry, National Institute of Diabetes and Digestive and Kidney Diseases, Bethesda, MD 20892 USA; 3 Regenerative Medicine Research Center, West China Hospital, Sichuan University, Keyuan Road 4, Gaopeng Street, Chengdu, Sichuan 610041 China

**Keywords:** Diseases, Endocrinology

Correction to: *Nutrition and Diabetes*

10.1038/s41387-017-0002-0 published online 19 December 2017

The original version of this article unfortunately contained a mistake in the affiliations. The correct affiliations are:

Hao Hong 1 Key Laboratory of Acupuncture and Medicine Research of Ministry of Education, Nanjing University of Chinese Medicine, Nanjing, Jiangsu 210023, China.

Zhen-Zhong Cui 2 Molecular Signaling Section, Laboratory of Bioorganic Chemistry, National Institute of Diabetes and Digestive and Kidney Diseases, Bethesda, MD 20892, USA.

Lu Zhu 2 Molecular Signaling Section, Laboratory of Bioorganic Chemistry, National Institute of Diabetes and Digestive and Kidney Diseases, Bethesda, MD 20892, USA.

Shu-Ping Fu 1 Key Laboratory of Acupuncture and Medicine Research of Ministry of Education, Nanjing University of Chinese Medicine, Nanjing, Jiangsu 210023, China.

Mario Rossi 2 Molecular Signaling Section, Laboratory of Bioorganic Chemistry, National Institute of Diabetes and Digestive and Kidney Diseases, Bethesda, MD 20892, USA.

Ying-Hong Cui 2 Molecular Signaling Section, Laboratory of Bioorganic Chemistry, National Institute of Diabetes and Digestive and Kidney Diseases, Bethesda, MD 20892, USA.

Bing-Mei Zhu 3 Regenerative Medicine Research Center, West China Hospital, Sichuan University, Keyuan Road 4, Gaopeng Street, Chengdu, Sichuan 610041, China.

