# Supplementary material for: Central IGF1 improves glucose tolerance and insulin sensitivity in mice
Source: Nutr Diabetes. 2017 Dec 19;7(12):2. doi: 10.1038/s41387-017-0002-0 (PMC5865549; doi:10.1038/s41387-017-0002-0)
Supplement: Supplementary file 1 — Fig-suppl [file 41387_2017_2_MOESM1_ESM.doc]

**Central IGF1 improves glucose tolerance and insulin sensitivity in mice**

Hao Hong^1^*, Zhenzhong Cui^3^*, Lu Zhu^3^, Shu-Ping Fu^1^, Mario Rossi^3^, Yinghong Cui^3^, Bing-Mei Zhu^1, 2§^

^1^Key Laboratory of Acupuncture and Medicine Research of Ministry of Education, Nanjing University of Chinese Medicine, Nanjing, Jiangsu, 210023, China

^2^Regenerative Medicine Research Center West China Hospital, Sichuan University, Keyuan Road 4, Gaopeng Street, Chengdu, Sichuan, 610041, China.

^3^Molecular Signaling Section, Laboratory of Bioorganic Chemistry, National Institute of Diabetes and Digestive and Kidney Diseases, Bethesda, MD, 20892, USA

**Supplementary Fig 1.**


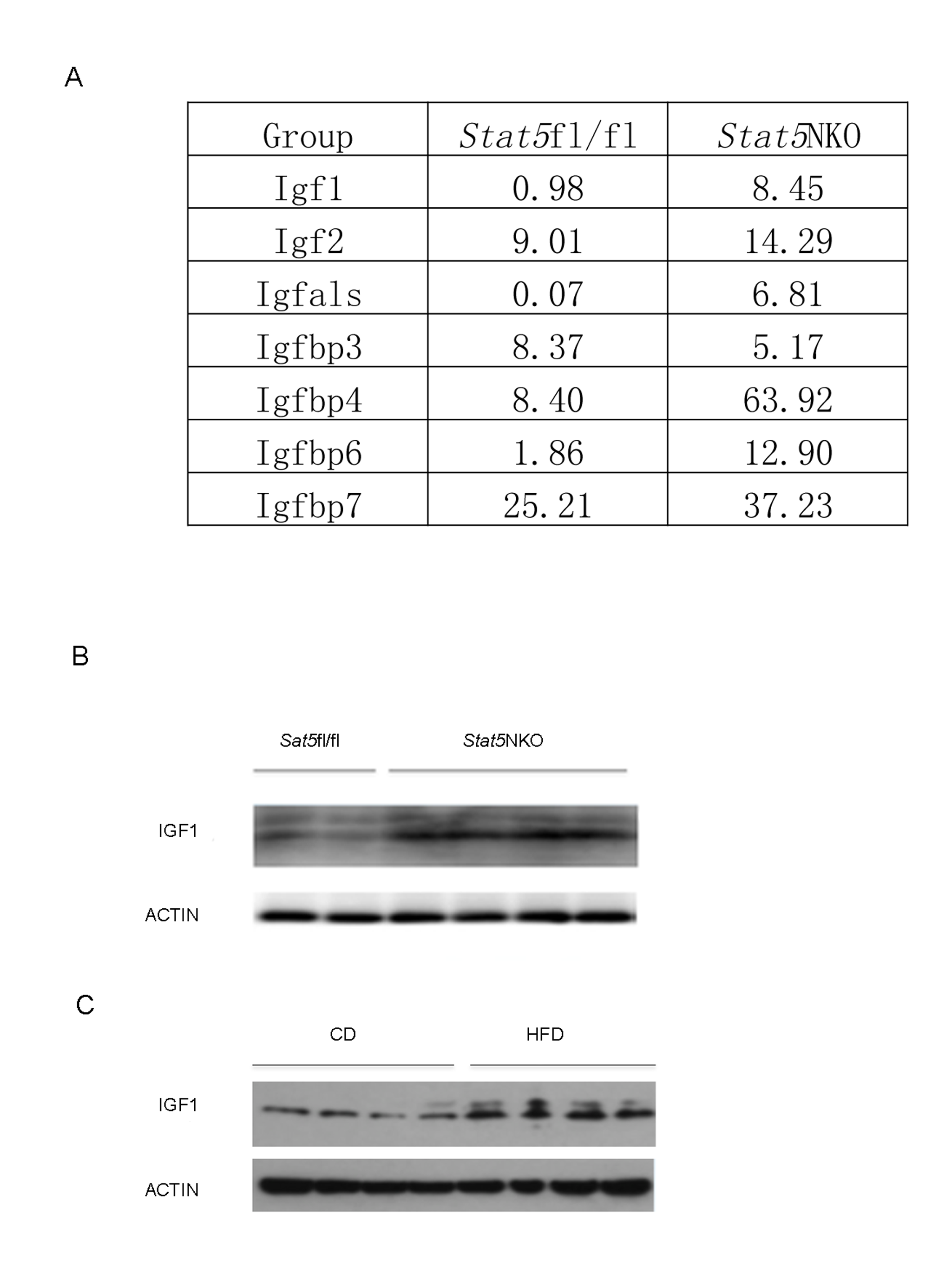


**Supplementary Fig 2.**

**
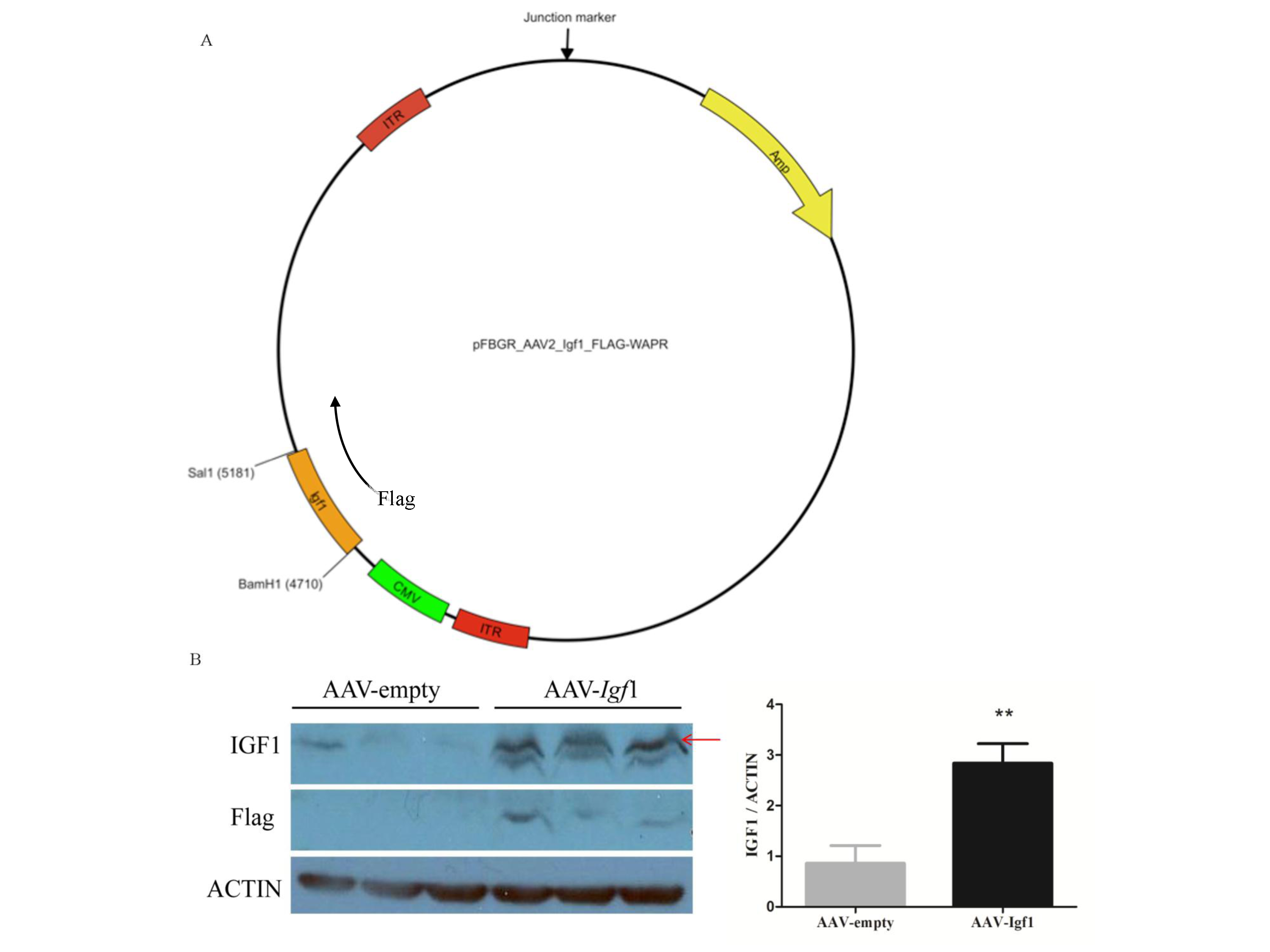
**

**Supplemental Fig.1. Igf1 is up-regulated in mice lacking Stat5 in the brain (*Stat5*NKO mice) and mice rendered obese by a high-fat diet (HFD).** (A) RNA-seq data were generated from hypothalamic RNA of *Stat5*NKO mice (n=3 or 4). (B) IGF1 expression was determined via immunoblotting using hypothalamic tissue from *Stat5*NKO and *Stat5*fl/fl control mice (n=2-4). (C) IGF1 expression was detected via immunoblotting using hypothalamic tissue of mice maintained on a HFD or regular chow diet (CD) (n= 4 per group). Mice were maintained on the HFD for 8 weeks.

**Supplemental Fig.2. Schematic diagram of the AAV-Igf1 construct (A) and detection of IGF1 expression (B).** IGF1 expression was detected via western blot in the ARC of wild-type mice injected with the AAV-*Igf*1 virus (n=3 per group). Mice (age: 12 weeks) were sacrificed to assess IGF1 over-expression one week after AAV injection.

**Supplementary Fig 3.**


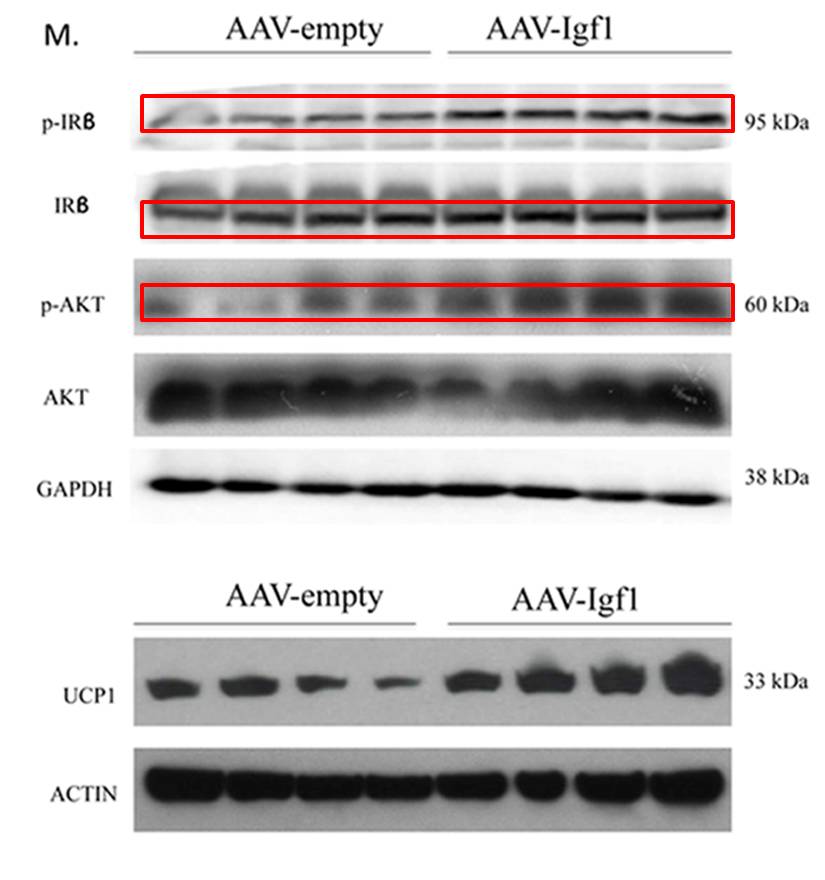


**Supplemental Fig.3.** Here we showed which band was quantified in Fig 4N, and p-IR or p-AKT were both quantified versus total IR or total AKT.

**Supplementary Table 1 The primer sequences for qRT-PCR.**

| **Mouse gene** | **Primer sequence** | **Amplicon (bp)** |
| --- | --- | --- |
| *GAPDH* | Forward: 5’ ACAGTCCATGCCATCACTGCC  Reverse: 5’ GCCTGCTTCACCACCTTCTTG | 266 |
| *Agrp* | Forward: 5’ CCCTCCCCAAGAATGGACTG  Reverse: 5’ GCAGTCCCAGCTCACAGATT | 103 |
| *Pomc* | Forward: 5’TAGATGTGTGGAGCTGGTGC  Reverse: 5’ACGTACTTCCGGGGGTTTTC | 169 |
| *Npy* | Forward: 5’GAACTCGGCTTGAAGACCCT  Reverse: 5’ GCATTGGTAGGATGGGTGGA | 120 |

**Supplementary Table 2 Antibodies used for Western blotting and immunofluorescence studies**

| **Antibody target** | **Source of antibody** | **Catalog #** |
| --- | --- | --- |
| IGF1 | Abcam | ab9572 |
| AKT | Cell Signaling Technology | 9272 |
| Phospho-AKT | Cell Signaling Technology | 9271 |
| IRβ | Cell Signaling Technology | 3025 |
| Phospho-IRβ | Cell Signaling Technology | 3023 |
| UCP1 | Abcam | ab10983 |
| GAPDH | Cell Signaling Technology | 2118 |
| β-ACTIN | Cell Signaling Technology | 3700 |
| Monoclonal ANTI-FLAGM2(for IF studies) | Sigma | F1804 |
| Anti-rabbit IgG, HRP-linked secondary antibody | Cell Signaling Technology | 7074 |
