# Supplementary material for: Central IGF1 improves glucose tolerance and insulin sensitivity in mice
Source: Nutr Diabetes. 2017 Dec 19;7(12):2. doi: 10.1038/s41387-017-0002-0 (PMC5865549; doi:10.1038/s41387-017-0002-0)
Supplement: Supplementary file 2 — Suppl-2 [file 41387_2017_2_MOESM2_ESM.doc]

// This macro, MeanSigPerPixel_ROI-bg.ijm, helps the user obtain an average signal/pixel, which

// which can often interpreted as (proportional to) total expression per sample.

// One channel in the rawly acquired image, the "measured channel", must contain the measured probe of interest,

// which has a linear relationship to the amount of signal (e.g. protein) one is trying to measure--

// e.g. fluorescently labeled antibodies.

// Other channels will be ignored.

// The mean signal is calculated from the chosen channel, after background subtraction,

// and after thresholding out non-cell or non-tissue pixels.

waitForUser("MACRO SUMMARY", "This macro, MeanSigPerPixel_ROI-bg.ijm, measures average signal/pixel, \n after background subtraction and thresholding out trashy areas.\n-->RECOMMENDED: BEFORE CONTINUING, SAVE CURRENT Results, ROIs, etc, and/or clear them.\n-->NOTE1: Some operations can be performed when macro is paused like this, without affecting macro.\n-->NOTE2: Use Esc key to stop macro at any time.\n--> CLick OK or press ENTER key to continue");

waitForUser("NEED IMAGE WITH BACKGROUND", "Select ORIGINAL image which has an area from which background can be calculated.\n You can do this from Window menu, or drag into Fiji.\n-->THEN click OK");

run("Duplicate...", "duplicate");

rename("BG_Image");

getDimensions(width, height, channels, slices, frames);

if (channels>1 || bitDepth()==24) {

run("Make Composite");

Stack.setDisplayMode("color");

waitForUser("PICK CHANNEL OF INTEREST", "Using scrollbar on bottom of the so-called BG_Image, make sure the image in the channel of interest is displayed.\n -->THEN click OK");

selectWindow("BG_Image");

Stack.getPosition(channel, slice, frame);

chosen_channel = channel;

}

run("Orange Hot");

run("Enhance Contrast", "saturated=0.5");

makeOval(200, 200, 40, 40);

waitForUser("CHOOSE BACKGROUND ROI", "Contrast has been enhanced and LUT changed to help you choose the appropriate background area.\n Grab circle ROI in its center with the left mouse button, and drag ROI to a background area.\n Global background per pixel will be assumed to be the statistical mode (most common value) of this ROI,\n and will then be subtracted from all measured images.\n--> Click on OK after choosing background ROI");

run("Set Measurements...", "modal display redirect=None decimal=0");

selectWindow("BG_Image");

run("Measure");

GlobalBG = getResult("Mode");

selectWindow("BG_Image");

run("Select None");

run("Subtract...", "value="+GlobalBG);

rename("BGSub_BGImage");

selectWindow("Results");

run("Close");

run("Orange Hot");

setAutoThreshold("Default dark");

setThreshold(20, 255);

call("ij.plugin.frame.ThresholdAdjuster.setMode", "Over/Under");

run("Threshold...");

waitForUser("SET THRESHOLD TO EXCLUDE BACKGROUND PIXELS", "(Btw, global background is "+GlobalBG+".)\nUsing the TOP SCROLLBAR IN THRESHOLD WINDOW, choose threshold that just barely turns background pixels blue without thresholding pixels in cells.\n -->THEN click OK");

selectWindow("BGSub_BGImage");

getThreshold(lowerThresh, upperThresh);

close("BGSub_BGImage");

for (s=1;s<=1000;s++) {

waitForUser("CHOOSE IMAGE TO MEASURE", "Open/select ORIGINAL multi-channel image that you wish to measure.\n You can use Fiji's Window menu if image is already open.\n--> THEN click OK");

BGSubImName = "BGsub_"+getTitle();

if (channels>1 || bitDepth()==24) {

run("Make Composite");

Stack.setDisplayMode("color");

Stack.setPosition(chosen_channel, 1, 1);

}

run("Select None");

run("Subtract...", "value="+GlobalBG);

rename(BGSubImName);

setTool("polygon");

setBackgroundColor(0, 0, 0);

call("ij.plugin.frame.ThresholdAdjuster.setMode", "Over/Under");

setThreshold(lowerThresh, upperThresh);

waitForUser("DELETE BAD AREAS NOT THRESHOLDED", "If necessary to remove trash from image, define ROI(s) to mask out by clicking polygon vertices on image, \nthen EDIT MENU-> CLEAR (DON'T FORGET!!.\n --> WHEN FINISHED DELETING BAD AREAS, click OK");

selectWindow(BGSubImName);

run("Set Measurements...", "mean integrated area_fraction limit display redirect=None decimal=1");

run("Select None");

run("Measure");

run("Tile");

selectWindow("Results");

}
